# Multiple-locus, variable number of tandem repeat analysis (MLVA) of the fish-pathogen *Francisella noatunensis*

**DOI:** 10.1186/1746-6148-7-5

**Published:** 2011-01-24

**Authors:** Øyvind J Brevik, Karl F Ottem, Are Nylund

**Affiliations:** 1 Department of Biology, University of Bergen, Post box 7800, N-5020 Bergen, Norway

## Abstract

**Background:**

Since *Francisella noatunensis *was first isolated from cultured Atlantic cod in 2004, it has emerged as a global fish pathogen causing disease in both warm and cold water species. Outbreaks of francisellosis occur in several important cultured fish species making a correct management of this disease a matter of major importance. Currently there are no vaccines or treatments available. A strain typing system for use in studies of *F. noatunensis *epizootics would be an important tool for disease management. However, the high genetic similarity within the *Francisella *spp. makes strain typing difficult, but such typing of the related human pathogen *Francisella tullarensis *has been performed successfully by targeting loci with higher genetic variation than the traditional signature sequences. These loci are known as Variable Numbers of Tandem Repeat (VNTR). The aim of this study is to identify possible useful VNTRs in the genome of *F. noatunensis*.

**Results:**

Seven polymorphic VNTR loci were identified in the preliminary genome sequence of *F. noatunensis *ssp. *noatunensis *GM2212 isolate. These VNTR-loci were sequenced in *F. noatunensis *isolates collected from Atlantic cod (*Gadus morhua*) from Norway (n = 21), Three-line grunt (*Parapristipoma trilineatum*) from Japan (n = 1), Tilapia (*Oreochromis *spp.) from Indonesia (n = 3) and Atlantic salmon (*Salmo salar*) from Chile (n = 1). The Norwegian isolates presented in this study show both nine allelic profiles and clades, and that the majority of the farmed isolates belong in two clades only, while the allelic profiles from wild cod are unique.

**Conclusions:**

VNTRs can be used to separate isolates belonging to both subspecies of *F. noatunensis*. Low allelic diversity in *F. noatunensis *isolates from outbreaks in cod culture compared to isolates wild cod, indicate that transmission of these isolates may be a result of human activity. The sequence based MLVA system presented in this study should provide a good starting point for further development of a genotyping system that can be used in studies of epizootics and disease management of francisellosis.

## Background

The intensive culturing of fish in artificially high population densities facilitates disease outbreaks [1,2]. Standard protocols for traditional fish health management, controlling and preventing diseases, have focused on vaccines, operational prophylactic measures and oral treatment using therapeutic agents [3]. However, in those cases where vaccines or treatments are not available, understanding the epizootiology becomes the key to prevent outbreaks and pathogen dispersal [1]. Using molecular tools for strain typing of pathogens, combined with biological and ecological knowledge of both the pathogen and the host, is a prerequisite in creating an epizootiological understanding which can be applied in management of diseases in both wild and cultured populations.

Historically, the Atlantic cod (*Gadus morhua*) has been an important marine resource in Norway, occurring naturally both as migratory and stationary populations [4-6]. These populations are divided into coastal and oceanic populations, based on phenotypic and genotypic traits. The Norwegian coastal cod population is considered to consist of several stationary sub populations dispersed along the Norwegian coast [7-12]. The oceanic cod in Norwegian waters can be divided into two populations, the North East Arctic cod and the North Sea cod, both with seasonally dependent migratory behavior. The oceanic and some of the coastal populations will, at certain times of the year, be present in at the same locations.

During the last decade cod has been a species of increasing significance and interest for the Norwegian aquaculture industry [13]. Cod is now intensively cultured with full control of all life-stages, except for the broodfish which are predominantly of wild origin [6,14]. The intensive production cycle of cod consists of three separate steps, where the first step starts with the fertilization of eggs from broodstock held in large land based tanks. The majority of broodfish consists of wild caught cod from both costal and oceanic populations. After hatching the fry is held in indoor tanks. The second stage is on-growth, where the fingerlings are transferred to net pens in the sea and held until they are moved to production sites. Mixing of populations from different broodstock companies occurs at the on-growth sites, i.e. before redistribution to production sites. Mixing of populations may also occur at the production sites due to limited availability of fish from the different on-growing sites. Several generations can be present at one production site. The different operational sites are dispersed along the Norwegian coast resulting in large scale movement of cultured cod between different parts of Norway [13,14].

In 2004 a *Francisella *species was isolated from farmed cod in Norway showing clinical signs of a chronic granulomatous infection in kidney, spleen, liver and heart [15]. The bacterium was initially characterized both as a species, *F. piscicida *(GM2212) [16,17], and later in the same year as a *F. philomiragia *subspecies, ssp. *noatunensis *(NCIMB 14265^T^) [18]. The *F. philomiragia *ssp. *noatunensis *was later elevated to the rank of species, *F. noatunensis *ssp. *noatunensis*, with *F. piscicida *as a heterotypic synonym [19-22]. Following the discovery, several annual outbreaks of francisellosis have been diagnosed with main foci in the western parts of Norway [23]. The bacterium has been detected in cultured fish from Rogaland to Nordland county (59-67°N), however, positive wild cod has only been identified south of Sogn og Fjordane county (61°N), indicating a southern natural reservoir of the bacteria [24]. After the discovery of *F. noatunensis *ssp. *noatunensis*, histological material collected in 1988 from a broodfish population in Hordaland county, has been stained positive with antiserum for the *F. noatunensis *ssp. *noatunensis *GM2212 isolate, suggesting the presence of the bacteria in Norwegian waters prior to the initial discovery [25].

During the last 10 years members of the genus *Francisella *have emerged as a global problem for aquaculture, causing mortality among a wide range of aquatic hosts. These *Francisella *isolates show high genetic similarities with *F. philomiragia *and with each other at the 16S rRNA-gene [15,26-32]. The close genetic relatedness has been confirmed with sequencing of several housekeeping genes showing identical sequences for Norwegian isolates [18]. A Chilean isolate from Atlantic salmon (*Salmo salar*) UA2660 [29] showed a high similarity to the Norwegian cod isolates (NCIMB 14265^T^/GM2212) and was therefore described as a new isolate of the *F. noatunensis *ssp. *noatunensis *[19,22]. Fish pathogenic *Francisella *isolates from Asia display high genetic similarity to UA2660 and NCIMB 14265^T^/GM2212 and constitute a separate subspecies, *F. noatunensis *ssp. *orientalis *(Ehime-1) [19,20,26]. *F. asiatica *(PQ1104) isolated from tilapia (*Oreochromis *sp.) in Costa Rica is identical with *F. noatunensis *ssp. *orientalis *(Ehime-1) with respect to phenotype and signature sequences (rRNA and housekeeping genes) [22].

Isolates of *F. noatunensis *ssp. *noatunensis *from Norway are identical when comparing 16S and housekeeping gene sequences [18,33], making genotyping and studies of epizootics difficult. If an epizootiological approach is to be applied for controlling and preventing dispersal of *Francisella *spp. in aquaculture, a tool for the identification of isolates is a necessity. Such a tool will make it possible to differentiate between wild endemic and anthropogenic dispersed strains. This approach also requires knowledge of the production history of cod and some knowledge of natural occurring strains in wild cod in the production areas. Targeting the housekeeping genes of bacterial fish pathogens has been applied for strain identification [34], but due to identical signature sequences this has not been possible to use on *F. noatunensis *[18,24,33]. Similar problems are known from epidemiological studies of human pathogenic bacteria like the *Mycobacterium tuberculosis, M. avium *ssp. *paratuberculosis, Bacillus anthracis, Yersinia pestis *and the related *F. tularensis *[35-38] where evolutionary or stochastic events have led to a dominance of highly fit clones making strain identification difficult [39].

By using genetic markers with higher mutational rates, like Variable Number of Tandem Repeat (VNTR), one can construct allelic profiles in Multiple Locus VNTR Analysis (MLVA) systems, making strain differentiation of clonal bacteria populations possible [40-42]. MLVA systems have already been applied in studies of *F. tularensis *isolates [43-46]. This study is an attempt to provide a sequenced based MLVA tool for studying the epizootics of *F. noatunensis *ssp. *noatunensis *in Norwegian cod farming. Our findings suggest that the MLVA system presented in this study is suitable for strain typing of *F. noatunensis *isolates from Norwegian cod, and the results indicate that there are only a few clades causing francisellosis outbreaks in Norwegian cod culturing.

## Methods

### Isolation of *Francisella noatunensis *ssp*. noatunensis*

**Figure 1:**
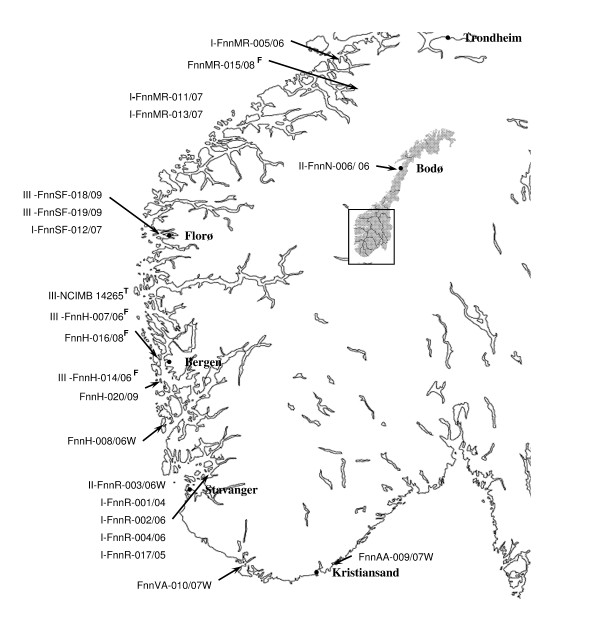
**The geographical location of sampling sites of the Norwegian *F. noatunensis *ssp. *noatunensis *isolates (n = 21)**. Accurate location of FnnMR-011/07 and FnnMR-013/07 is unknown. Isolates from production sites of cultured cod is predominantly of clade I and III. I = clade I, II = clade II, III = clade III, W = wild caught, broodfish^F^, type strain^T^

All isolates of *Francisella noatunensis *ssp*. noatunensis *(n = 22) included in this study were obtained from Atlantic cod (*Gadus morhua*) suffering from francisellosis, with the exception of the type strain NCIMB 14265^T ^[18] and the Chilean Atlantic salmon (*Salmo salar*) isolate UA2660 which were obtained from The National Collection of Industrial, food and Marine Bacteria (NCIMB) and Intervet Norbio AS, respectively (Table 1). The majority of isolates were collected from cultured populations of cod geographically dispersed along 1400 km of Norwegian coast line during the period from 2004 to 2009. Four isolates were obtained from wild-cod, from the counties of Aust-Agder, Vest-Agder, Rogaland and from the borders of Rogaland and Hordaland (Table 1). An overview of geographical origin of isolates is presented in Figure 1. Isolates of closely related *Francisella *spp., *F. notunensis *ssp. *orientalis *(n = 4) and *F. philomiragia *(n = 7), were also included in this study. Isolate FoJ-001/02 (Ehime-1) was kindly provided by Dr. Kamaishi, and isolates FoI-002/04, FoI-003/05 and FoI-004/07 from Indonesia were kindly provided by Intervet Singapore. Most *F. philomiragia *isolates were obtained from the culture collections Deutsche Sammlung von Mikroorganismen und Zellkulturen (DSMZ) and Culture Collection, University of Göteborg (CCUG), while strains 080107 and strain 1951[47,48] were kindly provided by Dr. Berrada and Dr. Friis-Møller, respectively. A complete overview of isolates and year of isolation, host and location is presented in Table 1.

**Table 1:** Sample information of the 33 strains of *Francisella *spp. included in the current study.

Strain name	Year	Host	Location
*F. noatunensis *subsp. *noatunensis*			

FnnR-001/04 (GM2212)	2004	Atlantic cod (*Gadus morhua*), farmed	Rogaland county
NCIMB 14265T	2005	Atlantic cod (*G. morhua*), farmed	Hordaland county
FnnR-017/05	2005	Atlantic cod (*G. morhua*), farmed	Rogaland county
FnnC-UA2660^A^	2006	Farmed Atlantic salmon (*Salmo salar*)	Region X Chile
FnnR-002/06	2006	Atlantic cod (*G. morhua*), farmed	Rogaland county
FnnR-003/06W	2006	Atlantic cod (*G. morhua*), wild caught	Rogaland county
FnnR-004/06	2006	Atlantic cod (*G. morhua*), farmed	Rogaland county
FnnMR-005/06	2006	Atlantic cod (*G. morhua*), farmed	Møre og Romsdalen county
FnnN-006/06	2006	Atlantic cod (*G. morhua*), farmed	Nordland county
FnnH-007/06 ^F^	2006	Atlantic cod (*G. morhua*), farmed	Hordaland county
FnnH-008/06W	2006	Atlantic cod (*G. morhua*), wild caught	Rogaland/Hordaland county
FnnH-014/06^F^	2006	Atlantic cod (*G. morhua*), farmed	Hordaland county
FnnAA-009/07W	2007	Atlantic cod (*G. morhua*), wild caught	Aust Agder
FnnVA-010/07W	2007	Atlantic cod (*G. morhua*), wild caught	Vest-Agder
FnnMR-011/07	2007	Atlantic cod (*G. morhua*), farmed	Møre og Romsdalen county
FnnSF-012/07	2007	Atlantic cod (*G. morhua*), farmed	Sogn og Fjordane county
FnnMR-013/07	2007	Atlantic cod (*G. morhua*), farmed	Møre og Romsdalen county
FnnMR-015/08^F^	2008	Atlantic cod (*G. morhua*), farmed	Møre og Romsdalen county
FnnH-016/08 ^F^	2008	Atlantic cod (*G. morhua*), farmed	Hordaland county
FnnSF-018/09	2009	Atlantic cod (*G. morhua*), farmed	Sogn og Fjordane county
FnnSF-019/09	2009	Atlantic cod(*G. morhua*), farmed	Sogn og Fjordane county
FnnH-020/09	2009	Atlantic cod (*G. morhua*), farmed	Hordaland county
*F. noatunensis *subsp. *orientalis*			

FnoJ-001/02 (Ehime-1)^B^	2002	Farmed Three-line grunt (*Parapristipoma trilineatum*)	Ehime prefecture, Japan
FnoI-002/04^C^	2004	Farmed tilapia (*Oreochromis *spp.)	Lake Toba, Indonesia
FnoI-003/05^C^	2005	Farmed tilapia (*Oreochromis *spp.)	Lake Toba, Indonesia
FnoI-004/07^C ^(Ind04)	2007	Farmed tilapia (*Oreochromis *spp.)	Lake Wadaslingtan, Indonesia
*F. philomiragia*			

FpDSM7535T	1959	Muskrat from Bear River refuge	Utah, USA
FpCCUG19701	1960	Water, from a river in Bear River refuge	Utah, USA
FpCCUG13404	1979	Human, bone marrow	Zurich, Switzerland
FpCCUG12603	1982	Human, abscess	Gøteborg, Sweden
Fp1951^D^	2003	Human, blood	Denmark
Fp080107 -I^E^	2007	Environmental sample direct isolation	Marthas Vineyard, USA
Fp080107 -II^E^	2007	Environmental sample after passage through a mice	Marthas Vineyard, USA

The table show source and geographical location of isolation for the *Francisella *isolates (n = 33) included in the current study. Norway is country of origin unless otherwise is stated.

Isolates were kindly provided by Intervet Norbio AS^A^, Dr. Kamaishi^B^, Intervet Singapore^C^, Dr. Friis-Møller^D ^and Dr. Berrada^E^

Isolated from broodfish^F^, Isolated in Chile^A^, W = wild caught, T = type strain

### Culture of *F. noatunensis *ssp. *noatunensis*

Isolations of *F. noatunensis *ssp. *noatunensis *were performed in the field or in our laboratory by streaking tissue (head kidney and/or spleen) from infected cod on agar plates. Initial isolations were performed using blood agar plates with 0.1% cysteine and 1% glucose as described by Nylund et al. (2006) with subsequent growth in B1817 broth for cryopreservation [33]. However, most isolations were performed using Cysteine Heart Agar (Difco) supplemented with 5% bovine blood (CHAB) as described in Olsen et al. (2006) with slight modifications. These included the addition of 50 μg ml^-1 ^of ampicillin, 50-100 μg ml^-1 ^of fungizone^® ^and additional cysteine to a final concentration of 0.2%. The ampicillin, fungizone^®^, bovine blood and cysteine were added to the CHAB at 60°C. All isolates were incubated at 20°C for 2-4 days (*F. philomiragia*) or two weeks (*F. noatunensis*). A Sanyo MIR-554 incubator was used for solid cultures whereas liquid culturing was performed in an Infors Unitron incubator at 250 rpm. Colonies from CHAB agar were dissolved in Eugon Broth (Difco) for cryopreservation and stored in liquid nitrogen.

### Genomic analysis

A preliminary genomic sequence from *Francisella noatunensis *ssp. *noatunensis *isolate GM2212 (= *F. piscicida*, DSM 18777^T^, CNCM-3511^T^, LMG24256^T^) was analyzed for the presence of tandem repeat regions using the software Tandem Repeats Finder [49]. This program locates and displays tandem repeats in DNA sequences. Loci with tandem repeats consisting of less than 15 nucleotides and more than four repeats were selected (Table 2). The preliminary genomic sequence of *F. noatunensis *ssp. *noatunensis *isolate GM2212 was generated using the 454 pyro-sequencing technology [50,51] in co-operation with Intervet AS/ASA.

**Table 2:** Attributes of the Variable Number of Tandem Repeat loci among *F. noatunensis *isolates.

Marker locus	GenBank Accesion no	Repeat sequence	Repeats in GM2212	Amplicon/repeat span	No. alleles
Fnn-VNTR1^NA^	GU385767	TTAAGGTA	7	195-219/5-8	3
Fnn-VNTR2	GU385768	AGTTATT	8	217-392/8-33	7
Fnn-VNTR3^NA^	GU385769	TAGAT	10	197-212/7-10	4
Fnn-VNTR4	GU385770	TTGTGG	10	311-437/10-31	11
Fnn-VNTR5^NA^	GU385771	AAAAGGTAT	5	285-312/2-5	2
Fnn-VNTR6	GU385772	CTG	10	295-307/8-12	3
Fnn-VNTR7	GU385773	TTTTA	5	404-435/1.8-5	4

GenBank Accession numbers refer to sequences of the *F. noatunensis *ssp. *noatunensis *GM2212 isolate. Repeat sequence, number of repeats and amplicon size is that of the GM2212 isolate.

Not amplified within *F. philomiragia *isolates^NA^

### DNA extraction

Total DNA from all collected strains and tissues was extracted using the DNeasy^® ^blood & tissue kit (Qiagen) as described by the manufacturer.

### PCR and amplification of VNTR loci

Based on results from the tandem repeats analysis, oligonucleotide primers flanking 11 potential VNTR loci was designed using the Vector NTI Suite 9.0 program package (InforMax Inc.). In addition, two pairs of flanking primers, VNTR1-2F/R and VNTR2-2F/R were designed for the purpose of nested PCR, as not all isolates were amplified using the initial primers. The oligonucleotide primers had Tm ranging from 40.9 to 55.4°C, and the annealing-temperature for each primer combination was therefore optimized using gradient PCR with the isolate GM2212 DNA as template (Table 3). The amplification was performed in a 50 μl reaction mixture containing 10 X buffer (Promega) including 1.5 mmol^-1 ^MgCl_2, _2.5 mM dNTP (Promega), 10 μmol^-1 ^of each primer (Invitrogen), 2 μl DNA and 0.6 U Thermal Ace™DNA polymerase (Invitrogen). Amplification was carried out in a Mastercycler gradient (Eppendorf) with denaturation at 95°C for 5 min; 35 cycles with denaturation at 95°C for 30 s, annealing at X°C (optimized annealing temperature Table 3) for 45 s and extension at 72°C for 1 min, followed by prolonged extension at 72°C for 10 min and a short storage at 4°C. All PCR-products were visualized using gel-electrophoresis. In cases where the initial PCR did not amplify a target sequence, several techniques were applied including nested- (VNTR 1 and 2), touchdown- and gradient-PCR. In the touchdown PCR, the amplification cycles were as follows; denaturation at 95°C for 5 min; followed by 6 times of 3 cycles of; denaturation at 94°C for 45 s, annealing for 45 s at 66-51°C with a reduction of 3°C at each cycle, and extension at 72°C for 1 min. The rounds of touchdown were immediately followed by 25 cycles with denaturation at 94°C for 45 s, annealing at 48°C for 45 s, extension at 72°C for 1 min; and a final extension at 72°C for 10 min before a short storage at 4°C. The gradient PCR amplification was as follows: denaturation at 95°C for 5 min; denaturation at 94°C for 45 s, 2 and 4°C below and over optimized annealing temperature (Table 3) for 45 s, extension at 72°C for 1 min; and a final extension at 72°C for 10 min before a short storage at 4°C.

**Table 3:** Primers used to amplify the VNTR regions included in the study.

Primer-name	Sequence	Tm°C	Target	Optimized Tm°	Amplicon size
Fnn-VNTR1F	ATCTTGGAAATTAACTACTTTC	46.1	VNTR no 1	55	211
Fnn-VNTR1R	ACCTTTTTCTACACCAATAG	47.2			
Fnn-VNTR2F	GTAAACGTAGTTTTTGGAAGTCCAT	53.7	VNTR no 2	54	217
Fnn-VNTR2R	GGATGGCAATCTTGTGTAAA	50.7			
Fnn-VNTR3F	CAAACCTTCATCTCCACTAC	50.4	VNTR no 3	50	212
Fnn-VNTR3R	TGCTCTTTTCCCTCTATATA	47.2			
Fnn-VNTR4F	AGTTTCATTTATCAGGTGAC	47.5	VNTR no4	54	311
Fnn-VNTR4R	AGACTAATAGCCTTCCAAAA	48.5			
Fnn-VNTR5F	CTGGACATTAGTATCAGGAT	48.3	VNTR no 5	55	312
Fnn-VNTR5R	GCAGTGGTAACAATTTTAAG	47.2			
Fnn-VNTR6F	GCTGTTGGAGATAGTAAATAATTGC	52.3	VNTR no 6	55	301
Fnn-VNTR6R	TTAGCTTCTTTAAGACCAAG	47.2			
Fnn-VNTR7F	CTTCTTCTCAACCATACCAG	50.1	VNTR no 7	54	439
Fnn-VNTR7R	ACAAGCATATAGACTTATATTGA	46.9			
Fnn-VNTR1-2F	AAATGTAGAGTTTCCATCCAAC	47.1	VNTR no 1	55	613
Fnn-VNTR1-2R	AGTCGTATTTCTGCTTCAATC	45.6			
Fnn-VNTR2-2F	CATGGGCTACTTTTGGAATATATAC	47.7	VNTR no 2	55	559
Fnn-VNTR2-2R	TCGGTTTGACTGATGTCATG	44.6			

Flanking primers were designed based on preliminary data of the whole genome sequence of *F. noatunensis *ssp. *noatunensis *isolate GM2212. The attributes of the primers in the study; primer sequence, optimized annealing temperature (Tm°) and amplicon size of the GM2212 isolate.

### Sequencing of VNTR loci

In order to identify repeat copy number variation among the isolates in question the resulting PCR-products were sequenced for each VNTR locus. PCR products were purified using the E.Z.N.A Cycle-Pure Kit (OMEGA) as described by the manufacturer. Sequencing was then performed in both directions using the PCR-primers (Table 3) and the ABI PRISM BigDye terminator chemistry (version 3.1) according to Applied Biosystems (ABI). All sequences were assembled using the Vector NTI Suite 9.0 program (InforMax Inc.). Possible genetic location of sequenced VNTR-loci was identified by GeneBank blastn searches performed using the complete amplicon sequence from each primer pair from either *F. philomiragia *or *F. noatunensis *ssp. *noatunensis *GM2212 isolate.

### Phylogenetic analysis

At each VNTR locus in a single taxon, the VNTR was coded as a discrete character (i.e. 1-9, A-H) based upon the specific number of repeats at the region in question. These allele profiles were used to construct a data matrix within the Mesquite System for Phylogenetic Analysis (Maddison, W. P. and D.R. Maddison. 2009. Mesquite: a modular system for evolutionary analysis. Version 2.71 http://mesquiteproject.org). The data-matrix was exported as a nexus file into PAUP 4.0 (Swofford, D. L. 2003. PAUP*. Phylogenetic Analysis Using Parsimony (*and Other Methods). Version 4. Sinauer Associates, Sunderland, Massachusetts)) for phylogenetic analysis using the neighbor-joining (NJ) distance method [52]. The phylogenetic NJ analysis was not bootstrapped as there were too few characters and taxa for correct estimation of sampling error.

### Test of VNTR stability

Since VNTR regions are considered to be one of the fastest evolving sequences of a genome, it was important to test the stability of the selected VNTR regions both *in vitro *and *in vivo *[53]. Mutations in VNTR regions have been shown during *in vitro *growth of both *Yersinia pestis *and *Escherichia coli *[54,55]. Therefore the effect of multiple passages of the *F. noatunensis *ssp. *noatunensis *GM2212 isolate on VNTR stability was tested as follows: a frozen first passage culture of the GM2212 isolate was streaked for isolation on CHAB, one single colony was transferred in each passage for 10 passages. DNA isolation, PCR and sequencing were performed on a colony from passage 10 as described above. PCR and sequencing were performed for all VNTR loci for verification of copy numbers (GM2212-P10 Table 4). The *in vivo *stability of *F. noatunensis *ssp. *noatunensis *GM2212 isolate was tested at different temperatures with material from challenge experiments described below. Copy numbers at each VNTR locus was confirmed through direct sequencing using DNA extracted from kidney tissues of challenged fish as templates (Table 4). Kidney tissue from one cod in each group challenged with GM2212, and held at 10 (F10), 14 (F14) and 18°C (F18), was sampled for extraction of bacterial DNA for direct sequencing. Tank conditions for all groups were as for the challenge experiment of the F10 group described in Nylund et al. (2006) [15]. The F14 and F18 group were bath challenged in 20 l of sea water containing a final concentration of 2.25 × 10^6 ^bacteria/ml. The bacterial suspension was prepared by inoculating several CHAB plates with the GM2212 isolate, harvesting + mixing of bacterial cultures in PBS and subsequently adding this to sea water. Tissues were sampled from the fish 90 days post challenge. The challenge experiments were approved by the Norwegian Animal Research Authorities (NARA).

**Table 4:** Allelic profiles from the stability testing.

Isolate	VNTR1	VNTR2	VNTR3	VNTR4	VNTR5	VNTR6	VNTR7
**GM2212**	**211nt - 7r**	**217nt - 8r**	**212nt - 10r**	**311nt - 10r**	**312nt - 5r**	**301nt - 10er**	**435nt - 5r**
GM2212-P10	*219nt - 8r*	217nt - 8r	212nt - 10r	311nt - 10r	312nt - 5r	301nt - 10er	435nt - 5r
Fc10	211nt - 7r	217nt - 8r	212nt - 10r	311nt - 10r	312nt - 5r	301nt - 10er	435nt - 5r
Fc14	211nt - 7r	217nt - 8r	212nt - 10r	311nt - 10r	312nt - 5r	301nt - 10er	435nt - 5r
Fc18	211nt - 7r	217nt - 8r	212nt - 10r	311nt - 10r	312nt - 5r	*395nt - 9er*	435nt - 5r

*In vivo *stability was shown on passage 10 (P10) of the GM2212 isolate and *in vitro *stability on infected cod held at 10 (Fc10), 14 (Fc14) and 18°C (Fc18). Results are displayed with number of nucleotides in repeat marker and numbers of repeats. Two shifts were observed in GM2212, VNTR-1 after 10 passages on CHAB agar at 20°C and in VNTR-6 after passage in cod held at 18°C. The original sequence is shown in bold, whereas the mutations are in italics.

Heterogeneous repeats with Single nucleotide polymorphism (snp), noted as nucleotide number in repeat sequence (nt) and at repeat of occurrence (r); e = 2 snp C-T nt1 r4 r6

### VNTR linkage disequilibrium

Linkage disequilibrium was calculated as standardized index of association (*I*_A_^S^), for all Norwegian *F. noatunensis *ssp. *noatunensis *isolates (n = 21) within the seven VNTR loci using the LIAN Linkage Analysis 3.5 online tool [56].

### Accession numbers

Sequences of all seven VNTR loci from the GM2212 isolate of *F. noatunensis *ssp. *noatunensis *were assigned GeneBank accession numbers as follows: VNTR-1: GU385767, VNTR-2: GU385768, VNTR-3: GU385769, VNTR-4: GU385770, VNTR-5: GU385771, VNTR-6: GU385772, VNTR-7: GU385773

## Results

### Variable Number of Tandem Repeats (VNTR) features

Sequencing of *Francisella noatunensis *and *F. philomiragia *isolates (Table 1) showed variation in seven of the 11 tested VNTR loci (Table 5). The remaining four VNTR loci were discarded as they did not get amplified from all *F. noatunesis *isolates or showed no allelic variation among the isolates included in this study. The seven VNTR loci used in this study had an allelic diversity ranging from two (VNTR-5) to 17 (VNTR-4) alleles in all isolates included (n = 33). The largest observed variation in allele sizes was found in VNTR-4 and spanned from one to 32 repeats in *F. philomiragia *isolates Fp080107-II and Fp1951, respectively. Primers for the seven informative VNTR loci (Table 3) provided PCR-products for all *F. noatunensis *ssp. *noatunensis *and *orientalis *isolates with the exception of VNTR-1 in isolate UA2660 and VNTR-3 in Ehime-1. VNTR-2 and 4 showed most variation among the *F. noatunensis *ssp. *noatunensis *isolates (n = 22), with seven different alleles in each, while only minor variation, two different alleles, was found in VNTR-5. The *F. noatunensis *ssp. *orientalis *isolates (n = 4) had identical alleles at VNTR-5 and -6, while VNTR-4 was shown to be the most polymorphic with four different alleles. The repeats and attributes of each VNTR locus in *F. noatunensis *can be viewed in table 2. VNTR-4, -6 and -7 were successfully amplified from all isolates of *F. philomiragia *included in this study. It was not possible to amplify VNTR-2 from the *F. philomiragia *isolate, Fp080107-I [48], and, with the exception of the amplification of VNTR-3 from DSM7535^T^, it was not possible to amplify VNTR-1, -3 and -5 from these isolates (Table 5). Among the *F. philomiragia *isolates most allele variation was seen in VNTR-4, whereas no variations were detected in sequences from VNTR-2 and -6. It is not known if the lack of a PCR product for a given VNTR locus is a result of primer mismatch or absence of the VNTR locus in question. Due to the preliminary status of the genome from which the VNTR loci were identified, the genomic locations of the loci remain unknown. Possible locations of the VNTR loci within the genome of *F. philomiragia *were identified by GeneBank blastn searches. The following matches were identified (Table 6); VNTR-2 locus (186 bp query) matched the intergenic segment located between the genes encoding the Bor lipoprotein and Nicotinamide Adenine Dinucleotide Phosphate -quinone reductase (1900473-1900533bp), VNTR-4 sequence (394 bp query) matched the intragenic segment in the *DNA-directed DNA polymerase *gene (695610- 695415), VNTR-6 sequence (327 bp query) matched the segment intragenically located in *50S ribosomal protein L10 gene *(1129799-1130125) ,VNTR-7 sequence (458 bp query) matched the intragenically located segment between and within the genes encoding the FTN_1059 hypothetical protein and trigger factor protein (1661303-1660846). No significant matches were identified for VNTR-3. VNTR-5 and -1 were not amplified from DSM 7535, but a blastn search was performed using these VNTR sequences obtained from *F. noatunensis *ssp. *noatunensis *GM2212 isolate. VNTR-5 displayed intragenic match for a gene encoding the FTN_0396 hypothetical protein (495433-496083) with 99% sequence-identity and E value of 3 × 10^-79^, while VNTR-1 showed no significant match to the *F. philomiragia *genome.

**Table 5:** Allelic profiles of the 33 *Francisella *isolates used in the study.

Allelic profile	Strain name	Fnn-VNTR1	Fnn-VNTR2	Fnn-VNTR3	Fnn-VNTR4	Fnn-VNTR5	Fnn-VNTR6	Fnn-VNTR7
								
	*F. noatunensis *ssp. *Noatunensis*							

	FnnUA2660^A^	NA	392nt - 33r	212nt - 10r	335nt - 14br	285nt - 2r	307nt - 12hr	405nt - 2ir
I	FnnR-001 -04 (GM2212) ^T^	211nt - 7r	217nt - 8r	212nt - 10r	311nt - 10r	312nt - 5r	301nt - 10er	435nt - 5r
I	FnnR-002 - 06	211nt - 7r	217nt -8r	212nt - 10r	311nt - 10r	312nt - 5r	301nt - 10er	435nt - 5r
I	FnnR-004 - 06	211nt - 7r	217nt - 8r	212nt - 10r	311nt - 10r	312nt - 5r	301nt - 10er	435nt - 5r
I	FnnR-017 - 05	211nt - 7r	217nt - 8r	212nt - 10r	311nt - 10r	312nt - 5r	301nt - 10er	435nt - 5r
I	FnnMR-005 - 06	211nt - 7r	217nt - 8r	212nt - 10r	311nt - 10r	312nt - 5r	301nt - 10er	435nt - 5r
I	FnnMR-011 - 07	211nt - 7r	217nt -8r	212nt - 10r	311nt - 10r	312nt - 5r	301nt - 10er	435nt - 5r
I	FnnSF-012 - 07	211nt - 7r	217nt - 8r	212nt - 10r	311nt - 10r	312nt - 5r	301nt - 10er	435nt - 5r
I	FnnMR-013 - 07	211nt - 7r	217nt - 8r	212nt - 10r	311nt - 10r	312nt - 5r	301nt - 10er	435nt - 5r
II	FnnR-003 - 06W	211nt - 7r	217nt - 8r	207nt - 9r	311nt - 10r	312nt - 5r	301nt - 10er	435nt - 5r
II	FnnN-006 - 06	211nt - 7r	217nt - 8r	207nt - 9r	311nt - 10r	312nt - 5r	301nt - 10er	435nt- 5r
III	FnnH-007 - 06 ^F^	219nt - 8r	259nt - 14r	202nt - 8r	341nt - 15r	312nt - 5r	301nt - 10er	430nt - 4r
III	NCIMB 14265^T^	219nt - 8r	259nt - 14r	202nt - 8r	341nt - 15r	312nt - 5r	301nt - 10er	430nt - 4r
III	FnnH-014 - 06^F^	219nt - 8r	259nt - 14r	202nt - 8r	341nt - 15r	312nt - 5r	301nt - 10er	430nt - 4r
III	FnnSF-018 - 09	219nt - 8r	259nt- 14r	202nt - 8r	341nt - 15r	312nt - 5r	301nt - 10er	430nt- 4r
III	FnnSF-019 - 09	219nt - 8r	259nt - 14r	202nt - 8r	341nt - 15r	312nt - 5r	301nt - 10er	430nt - 4r
	FnnH-008 - 06W	195nt - 5r	217nt - 8r	202nt - 8r	311nt - 10r	312nt - 5r	301nt - 10er	435nt - 5r
	FnnMR-015 - 08^F^	195nt - 5r	259nt - 14r	197nt - 7r	437nt - 31r	312nt - 5r	301nt - 10er	435nt - 5r
	FnnH-016 - 08^F^	219nt - 8r	287nt - 18r	197nt - 7r	359nt - 18r	312nt- 5r	295nt - 8er	435nt - 5r
	FnnAA-009 - 07W	211nt - 7r	294nt - 19r	197nt - 7r	347nt - 16r	312nt - 5r	301nt - 10er	435nt - 5r
	FnnVA-010 - 07W	211nt - 7r	308nt - 21r	197nt - 7r	329nt - 13r	312nt - 5r	301nt - 10er	435nt - 5r
	FnnH-020 - 09	195nt - 5r	280nt - 17r	207nt - 9r	437nt - 31r	312nt - 5r	301nt - 10er	435nt - 5r
								
	*F. noatunensis *ssp. *orientalis*							

	FnoJ-001 - 02 (Ehime-1)	195nt - 5r	217nt - 8r	NA	317nt - 11dr	285nt - 2r	301nt - 10fr	405nt - 2ir
	FnoI-002 - 04 1368	211nt - 7r	392nt - 33r	212nt - 10r	365nt - 19dr	285nt - 2r	301nt - 10fr	404nt - 1.8r
	FnoI-003 - 05 1436	211nt - 7r	217nt - 8r	212nt - 10r	329nt - 13dr	285nt - 2r	301nt - 10fr	404nt - 1.8r
	FnoI-004 - 07 2070	211nt - 7r	217nt - 8r	212nt- 10r	371nt - 20dr	285nt - 2r	301nt - 10fr	404nt - 1.8r
								
	*F. philomiragia*							

	FpDSM7535T	NA	175nt - 2.8br	182nt - 4c	401nt - 25dr	NA	301nt - 10gr	404nt - 1.8r
	FpCCUG19701	NA	175nt - 2.8br	NA	395nt - 24dr	NA	301nt - 10gr	405nt - 2r
	FpCCUG13404	NA	175nt - 2.8br	NA	311nt - 10dr	NA	301nt - 10gr	404nt - 1.8r
	FpCCUG12603	NA	175nt - 2.8br	NA	407nt - 26dr	NA	301nt - 10gr	425nt - 3r
	Fp1951	NA	175nt - 2.8ar	NA	443nt - 32r	NA	301nt - 10gr	404nt - 1.8r
	Fp080107 -I	NA	Na	NA	431nt - 30dr	NA	301nt - 10gr	405nt - 2r
	Fp080107 -II	NA	175nt - 2.8ar	NA	257nt - 1ar	NA	301nt - 10gr	404nt - 1.8r

Identical allelic profiles are assigned Greek numerals I-III. Allelic profiles are presented as number of nucleotides in repeat marker (nt) and numbers of repeats (r). Identical profiles occur for several isolates from cod culturing, profile I (FnnR-001-04 to FnnMR-013-07), profile II (FnnR-003-06W and FnnN-006-06) and profile III (FnnH-007-06 to FnnSF-019-09)

Isolated in Chile^A^, Isolated from broodfish^F^, type strain^T^, W = wild caught, NA = Not Amplified with primer sets

Heterogeneous repeats with Single Nucleotide Polymorphism (snp), noted as nucleotide number in repeat sequence (nt) and at repeat of occurrence (r); a = snp G-A nt1 r1 r3 b = snp. G-A nt1 r3, c = snp G-A nt3 r4, d = snp T-C nt1 from r2, e = 2 snp C-T nt1 r4 r6, f = 2 snp C-T nt1 r5 r7, g = 2 snp C-T nt1 r6 r8, h = 2 snp C-T nt1 r8 r10, i = snp T-C nt4 r1

**Table 6:** Location of Blastn matches from Variable Number of Tandem Repeat -sequences from *F. philomiragia *DSM7535^T^.

Marker locus	BP in query	Identity	Location	Adjacent genes	E-value
Fnn-VNTR2	186	96%	Intergenic (1900473-1900533)	*Bor lipoprotein/NADPH-quinone reductase*	7 × 10^-28^
Fnn-VNTR4	394	97%	Intragenic (695610- 695415)	*DNA-directed DNA polymerase*	1 × 10^-88^
Fnn-VNTR5 ^A^	333	99%	Intragenic (495433-496083)	*FTN_0396 hypothetical protein*	3 × 10^-79^
Fnn-VNTR6	327	100%	Intragenic (1129799-1130125)	*50S ribosomal protein L10*	7 × 10^-170^
Fnn-VNTR7	458	97%	Intragenic (1661303-1660846)	*FTN_1059 hypothetical and trigger factor protein*	0

The Blastn search was performed using the complete amplicon of the primer pairs. All locations were identified in *F. philomiragia *ATCC25017^T ^(Accession number CP000937). Sequences were derived from *F. philomiragia *DSM7535^T ^unless otherwise stated.

VNTR-sequence obtained from *F. noatunensis *ssp. *noatunensis *GM2212^A^

### Allele profiles

Variation in the VNTR loci was revealed by sequencing, and these data were used to create allele profiles for all isolates. Lack of amplifiable VNTR locus was interpreted as a separate "missing" character. A complete overview of the allele profiles is presented in Table 5. Analysis of the seven VNTR loci revealed a total of 10 unique allele profiles among the 22 *F. noatunensis *ssp. *noatunensis *isolates. Three of the profiles contained more than one isolate and these were termed profile I (n = 8), profile II (n = 2) and profile III (n = 5); all isolates included in these profiles were collected from farmed sites with the exception of one wild isolate in profile II.

Profile I included the following isolates from farmed cod: a) four isolates collected in the period of 2004-06 from the Rogaland county, b) one isolate from Sogn and Fjordane county (2007) and c) three isolates from Møre and Romsdal county (2006-07). The two isolates in profile II were collected from Rogaland (wild cod) and Nordland (farmed cod) in 2006. A total of five isolates were included in profile III: a) three isolates, including two originating from broodfish populations, collected in Hordaland county during the period 2005-06, and b) two isolates, one sampled from farmed and one from a wild/possible escaped farmed cod in Sogn og Fjordane county in 2009. The remaining six cod isolates, including three isolates from wild cod, two from broodstock populations (usually caught as wild cod) and one from a production site, all possessed unique allele profiles. The two isolates from wild cod in Aust- and Vest Agder counties share allele profiles at five out of seven loci.

The allele profile of the Chilean isolate from Atlantic salmon, UA2660, had a unique profile sharing only one VNTR locus with the other isolates of *F. noatinensis *ssp. *noatunensis*.

All seven VNTRs were obtained from the isolates of *F. noatunensis *ssp. *orientalis *(n = 4) with the exception of VNTR-1 from Ehime-1. None of the isolates had identical allele profiles and the allele profile of the Japanese isolate Ehime-1, from farmed Three-line grunt (*Parapristipoma trilineatum*), was distinct from the three Indonesian isolates from farmed tilapia (*Oreochromis *spp.). None of the *F. philomiragia *isolates (n = 7) had identical allelic profiles.

### Phylogenetic relationship

**Figure 2:**
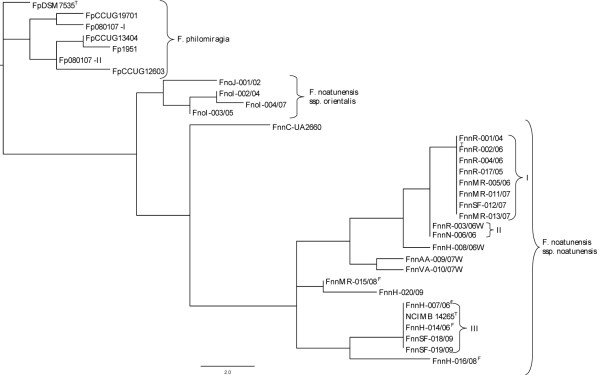
**The relationship of the isolates included in this study**. Relationship among the 33 *Francisella *isolates based on allelic differences at seven VNTR loci. This unrooted ultrametric NJ tree show known topography a) between the species of *F. philomiragia *(Fp) and *F. noatunensi*s, b) between the subspecies *F. noatunensis *ssp. *orientalis *(Fno) and *F. noatunensis *ssp. *noatunensis *(Fnn) and c) among the *F. noatunensis *ssp. *noatunensis *isolates from Norway and Chile (FnnC-UA2660). Two previously unrecognized clades are evident within the Norwegian Fnn isolates. Bootstrap values were not calculated as there were too few characters and taxa in the dataset for correct estimation of sampling error W = wild caught, broodfish^F^, type strain^T^

The NJ phylogenetic analysis using the seven informative VNTR loci from the 33 *Francisella *spp. isolates showed a subdivision of the *F. noatunensis *ssp. *noatunensis *isolates (Figure 2). The majority of the isolates from outbreaks of francisellosis in farmed cod group as two distinct clades. These clades were named clade I and III, as they correlate to the allele profiles (Table 5). The phylogenetic analysis also separates the Chilean UA2660 isolate from the Norwegian *F. noatunensis *ssp. *noatunensis *isolates, and differentiated among the few isolates of *F. noatunensis *ssp. *orientalis*, included.

### Stability of VNTR loci

Examination of the stability of the VNTR loci revealed changes in repeat numbers within two VNTRs of the *F. noatunensis *ssp. *noatunensis *GM2212 isolate (Table 4). A shift in VNTR-1 was observed after 10 passages on Cysteine Heart Agar with Blood (CHAB) at 20°C. This mutation consisted of the addition of one repeat, increasing the size from 7 to 8 repeats. There was also a change in VNTR-6 after passage of isolate GM2212 in cod held at 18°C. The number of repeats was reduced from 10 to nine. The VNTRs did not change when the isolate GM2212 was passed through cod held at 10 and 14°C. These results were verified by a repeating round of PCR and sequencing. The remaining VNTRs were not affected in this test.

### Linkage disequilibrium

To avoid effects from host and geographical separation the linkages disequilibrium was calculated for *F. noatunensis *ssp. *noatunensis *isolates (n = 22) only. The linkages disequilibrium was shown to be significant in the LIAN 3.5 analysis, indicating a clonal population structure for the cod isolates. Standardized *I*_A_^S ^was calculated to 0.3925 at a significance of P_para _= 3.69 × 10^-126^.

### Epizootiological data of isolates

There are several factors linking the isolates within the *F. noatunensis *clades (Figure 2, Table 5). Clade I consists of eight isolates where FnnR-001-04, -002-06, 004-06 and 017-05 were obtained from cod at one production site in Rogaland county during several outbreaks in the period of 2004-06 (Figure 1). These isolates differ from an isolate (FnnH-016-08) obtained from one of the broodfish company supplying the site. On-growth sites were not involved. Three isolates FnnMR-011-07, -013-07 and -005-06 of clade I were from another county (Møre og Romsdal), and two of these were obtained from cod with no traceable history. The third isolate FnnMR-005-06 was obtained from a site supplied by the same broodfish company as the host for isolate FnnSF-012-07, which also belongs to clade I. The latter isolate comes from another county, Sogn og Fjordane. The host for FnnMR-005-07 was kept at an on-growing site before transportation to the production site in Møre og Romsdal. Clade III consists of five isolates, three from Hordaland (FnnH-007-06, -014-06, NCIMB 14265T) and two from Sogn of Fjordane county (FnnSF018-09,-019-09) (Figure 1 and Figure 2). The latter two were sampled in the same fjord, FnnSF018-09 was isolated from farmed cod at a production site, while Fnn-019-09 was isolated from a wild caught cod. The wild caught cod had pellets in the gut and a morphology suggesting that it originated from a production site which lost fish during a francisellosis outbreak in 2009. Of the last three isolates from clade III, one (NCIMB 14265T) was isolated from cod at a production site in Hordaland, while FnnH-007-06 and FnnH-014-06 was isolated from broodfish populations located at two sites in the same county. This broodfish company was one of the suppliers of cod to the production site in Sogn and Fjordane (FnnSF018-09) and to one production site in Hordaland (NCIMB 14265T). It is not known if the cod at the two sites, Sogn and Fjordane and Hordaland (FnnSF018-09 and NCIMB 14265T), were offspring from the broodfish populations where FnnH-007-06 and FnnH-014-06 were isolated.

## Discussion

### Variable Number of Tandem Repeats (VNTR) system

Whole genome sequencing of bacteria has presented new opportunities for identification of new genetic markers for separation of isolates. One such marker system can be found by looking at VNTRs, i.e., single locus sequences with short DNA repeats [40,57]. Micro satellites, a subset of VNTRs, with repeat motifs of nine bp or less are often targeted due to higher mutational rate [58,59]. However, such hyper variability, that may occur within some VNTRs, would complicate determination of genetic relationships among strains using this method, and hence, its use in phylogenetics may not be ideal [45,54]. The mechanisms behind the length variation in micro satellites is that of Slipped-Strand Mispairing (SSM) during DNA polymerase mediated DNA duplication. However, mutations involving indels of large copynumbers have been shown consistent with recombination-mediated events [54,59,60]. In *Escherichia coli*, an average mutational rate of 6.4 × 10^-4 ^was calculated over 28 VNTRs, and the rate seems to be dependent on intrinsic properties such as numbers of repeats [54]. Variation in copy numbers at VNTR loci of certain sizes may affect the efficiency of promoters, thus affecting the coding potential of genes dependent on the genomic locations and the indels of repeats [58,61-63]. For differentiation of bacterial isolates several VNTR loci are combined in a Multiple Locus VNTR Analysis (MLVA), a well established tool for epidemiological studies of bacteria with highly fit clonal dominance [40-42,44,58]. One such bacteria is *Francisella tularensis*, the agent for tularaemia [64]. This species consist of clonal geographical subspecies [39] that can be separated with the help of VNTR markers [43-46,65]. A similar approach could also be the solution for genotyping of *Francisella *isolates belonging to fish pathogenic *Francisella *spp.

Most MLVA systems applied in epidemiology are performed by multiplexing PCR-assays where VNTR size is deduced by capillary electrophoresis allowing rapid screening of large datasets [45]. Due to the relatively few VNTR-loci included and that there were only 33 isolates available for analysis in this study, sequencing was selected as a method for repeat number verification. The sequencing also detected single nucleotide polymorphisms in addition to allele size. The future potential for use of multiplex PCR assays with dyed primers and determining allele size by capillary electrophoresis is still an option and is recommended for larger datasets. This study presents a sequence based MLVA system consisting of seven VNTR loci identified in the *F. noatunensis *ssp. *noatunensis *GM2212 isolate. When applied to 33 *Francisella *strains including Norwegian and Asian isolates of *F. noatunensis*, the MLVA provided the best resolution shown for the fish-pathogenic *Francisellae *so far. Due to the preliminary genome status of the *F. noatunensis *ssp. *noatunensis *GM2212 isolate a complete VNTR locus search has not been performed, thus leaving the possibility for unidentified markers yet to be discovered. Nonetheless strain typing of *F. tularensis *isolates has been achieved with the use of as few as two and six VNTR markers [43,65].

The exact location of the seven VNTR markers in the *F. noatunensis *ssp. *noatunensis *is not known, however, blast searches indicate both intra- and intergenic location if the positions are homologous with that of the *F. philomiragia *ATCC25017^T ^isolate (Table 6).

The discriminatory power of the VNTR loci used in this study, was not calculated as a Simpson`s index of diversity. The reason for this is that the dataset does not fulfill the criteria of a test population proposed by van Belkum et al. (2007) [53], as isolates are predominately from outbreaks in cultured populations of cod with potential epizootic connections. The VNTR markers had a typeability of 100% among the Norwegian isolates (n = 21), and with the exception of VNTR-1 and -3 for the UA2660 and Ehime isolate respectively, all the VNTR markers for *F. noatunensis *(n = 26) were amplified, indicating the usability of the typing system. Stability testing showed variability within VNTR-1 and -6 from *F. noatunensis *ssp. *noatunensis *GM2212 isolate when grown *in vitro *for 10 passages at 20°C and *in vivo *in cod at 18°C. Such single repeat changes are shown to occur in 80% of mutational events with equal chance of being an insertion or a deletion [54]. The sampled size was insufficient to assess the potential of hyper variable sites and mutation rates in the *F. noatunensis *ssp. *noatunensis *GM2212 isolate. VNTR mutational rates can differ across loci [54,55], and could be a problem in studies of phylogenetics and epizootics. Stability and introduction of variation could be a result of bacterial phase variation and thus a result of environmental factors such as nutritional substances present in the growth medium, temperature effects [60,63,66], or just simply be stochastical events. However, four isolates (FnnR-001-04, -002-06, 004-06, 017-05) from different francisellosis outbreaks at the same site during a two year period showed identical allelic profiles indicating field stability and epizootic coherence. The NJ dendrogram presented in this study should not be viewed as a phylogenetic analysis as the use of VNTRs and especially highly variable VNTRs create noise in the phylogenetic signal. The stability testing in the study shows that this may be the case of the VNTR-1 and VNTR-6. There are also too few isolates and characters (VNTRs) included in the dataset to attempt a proper analysis of genetic relationships.

### *F. noatunensis *strains

A total of nine allele profiles were identified among the Norwegian *F. noatunensis *ssp. *noatunensis *isolates (n = 21). Three of these contained more then one isolate (profile I n = 8, profile II n = 2 and profile III n = 5) and were identified using allelic profiles and visualized in the NJ dendrogram. The remaining six isolates represent unique strains of which five originated from wild cod and one from farmed cod (FnnH-020/09). Although the dataset of Norwegian isolates is small the trend is evident with only a few isolates occurring in outbreaks of francisellosis in cod culture, and a high diversity of strains among the isolates from wild cod. The Chilean isolate (UA2660) and the Norwegian cod isolates have previously been reported to be highly similar based on similarities in 16S sequence (99.8%) and in five housekeeping genes (Average Nucleotide Identity = 99.5%) [22,29]. However, according to the VNTR analysis performed in this study, the UA2660 isolate from salmon differs at six of seven markers compared to the Norwegian *F. noatunensis *ssp. *noatunensis *isolates from cod, clearly separating them into two distinct branches in the NJ dendrogram. The clear separation of these isolates most likely reflects geographic origin and the ecological differences between them (host-niche, freshwater vs. marine environment). The allele profiles of the four *F. noatunensis *ssp. *orientalis *isolates were unique, separating them into four different genotypes. Although the sampling set is small it correctly reflects the geographical origin and, possibly, also the variation in niche. There was no clear pattern in the clustering of the *F. philomiragia *isolates.

Care is needed if phylogenetic relationships are to be estimated based on VNTRs [54,58], however the deeper nodes in the dendrogram (Figure 2) a) between the species of *F. philomiragia *and *F. noatunensi*s, b) between the subspecies *F. noatunensis *ssp. *orientalis *and *F. noatunensis *ssp. *noatunensis *and c) among the *F. noatunensis *ssp. *noatunensis *isolates of Norway (NCIMB 14265T) and Chile (UA2660), were similar to the tree topology obtained based on analysis of 16S rRNA gene and datasets from housekeeping genes [19,22].

### Epidemiology of the *F. noatunensis*

Considering the results of the present study some reflections regarding the epizootics of the *F. noatunensis *are possible. However, it is necessary to acknowledge the shortcomings of the dataset as it covers about 40% of the official diagnosed outbreaks of francisellosis in Norway and that it only includes 4 wild isolates collected in different counties.

Generally, the low allelic diversity among the isolates collected from cultured cod compared to the isolates from wild cod, indicate that the spreading could be a result of human activity. This is also supported by the fact that the production cycle of cod in Norway involves transport of farmed cod along most of the Norwegian coast. Similarly, human activity has also been suggested as a cause for the geographical distribution of clonal isolates of *Bacillus anthracis *[37].

Although the VNTR markers provide a good starting point for studies of epizootics it may still be difficult to identify sources of infection as fish from different populations are mixed during the production, and, it is very difficult to avoid interactions between cultured and wild fish species. In the case of clade I with the isolates FnnR-001-04, -002-06, 004-06 and 017-05, the new generations of cod used for restocking after initial outbreak were probably infected by the initial isolate in the population at that site. The source of the initial infection was most likely cultured cod, since isolates belonging to the same genotype have been found at production sites in other counties, while the wild type isolate (FnnR-003/06W) from the same county belonged to another clade.

In clade III, the isolates from a known broodfish supplier were identical to isolates from production sites where the aforementioned broodfish company was one of the suppliers of fish. Hence, all of these cases could be a result of transmission through movement of offspring from broodfish. However, the situation is complicated by the fact that the sites used for on-growth are unknown. Vertical transmission would greatly impact the disease management and needs to be addressed in further studies.

## Conclusions

We present the first VNTR analysis of fish pathogenic *Francisellae*. Seven polymorphic microsatellites were identified in a partial genome sequence of the *F. noatunensis *ssp. *noatunensis *GM2212 isolate. A sequence based MLVA system of these seven VNTR-loci was applied to 33 aquatic *Francisella *isolates, including both *F. philomiragia *(n = 7) and *F. noatunensis *isolates (n = 26). All VNTRs were amplified in the *F. noatunensis *isolates, with the exception of VNTR-1 in UA2660 and VNTR-3 in Ehime-1. Among the Norwegian *F. noatunensis *ssp. *noatunensis *isolates (n = 21), including isolates from both farmed (n = 17) and wild cod, a total of nine allelic profiles were identified. The majority of farmed isolates were divided into two allelic profiles, indicating low allelic variation in isolates from outbreaks in cod culture compared to isolates collected from wild cod. The allelic profile of the Chilean *F. noatunensis *ssp. *noatunensis *isolate reflected the geographical and host divergence when compared to Norwegian cod isolates. All *F. noatunensis *ssp. *orientalis *isolates (n = 4) show a unique allelic profile. The four VNTRs amplified from *F. philomiragia *provided a unique allelic profile for all these isolates. The results show that this MLVA system should provide a good starting point for future studies of epizootics of *F. noatunensis*.

## Authors' contributions

ØJB was responsible for carrying out this study and was the main contributor in writing the manuscript. KFO identified the VNTR loci in the draft genome, contributed to project design and writing. AN coordinated the project, contributed to project design and reviewed all drafts of the manuscript All authors read, commented on and approved the final manuscript.
